# Kangai Injection, a Traditional Chinese Medicine, Improves Efficacy and Reduces Toxicity of Chemotherapy in Advanced Colorectal Cancer Patients: A Systematic Review and Meta-Analysis

**DOI:** 10.1155/2019/8423037

**Published:** 2019-07-15

**Authors:** Siqi Huang, Weijun Peng, Dan Mao, Shaofan Zhang, PanPan Xu, PengJi Yi, Sifang Zhang

**Affiliations:** ^1^Department of Integrated Traditional Chinese and Western Internal Medicine, The Second Xiangya Hospital of Central South University, Changsha, Hunan 410000, China; ^2^Department of Oncology, Yueyang Hospital of Traditional Chinese Medicine, Yueyang, Hunan 414000, China

## Abstract

**Objective:**

To systematically review whether the Kangai injection (KAI), which is commonly used traditional Chinese medicine, can improve the clinical efficacy of chemotherapy and relieve adverse reactions of chemotherapy in advanced colorectal cancer (CRC) patients.

**Methods:**

A comprehensive literature search was performed in three English and three Chinese electronic databases until March 2019. The literature was screened by EndNote X8 and data were analysed by RevMan5 and Stata12.0.

**Results:**

This meta-analysis consisted of twenty-eight studies, of which 2310 cases were reported. Among the 2310 cases, 1207 cases were treated with KAI combined with chemotherapy and 1103 cases were treated with chemotherapy alone. The results showed that KAI combined with chemotherapy significantly improved tumor response (Risk Ratio (RR) =1.32; 95% confidence interval (CI): 1.22-1.43;* p*<0.00001); Karnofsky performance status (KPS score) (Risk Ratio (RR) =1.48; 95% CI: 1.36-1.60;* p*<0.00001); reduced adverse drug reactions (ADRs) such as nausea and vomiting (OR =0.31; 95% CI: 0.24-0.41;* p *<0.00001), diarrhea (OR =0.36; 95% CI: 0.25-0.52;* p*<0.00001), leukopenia (OR =2.97; 95% CI:2.27-3.88;* p*<0.00001), thrombocytopenia (OR =0.53; 95% CI: 0.38-0.74;* p*<0.0002), liver dysfunction (OR =0.29; 95% CI: 0.20-0.44;* p*<0.00001), neurotoxicity (OR =0.51; 95% CI: 0.36-0.71;* p *= 0.0004); increased immune function (CD3^+^: MD=6.34; 95% CI: 5.52-7.16;* p *< 0.00001, CD4^+^: MD=-5.99; 95% CI: 5.20-6.78;* p *< 0.00001; and CD4^+^/CD8^+^: MD=0.34; 95% CI: 0.14-0.54;* p *< 0.0009), and prolonged survival time (OR =1.77; 95% CI: 1.25-2.50;* p = *0.001). Renal dysfunction caused by chemotherapy was not affected by KAI treatment (Odds Ratio (OR) =0.53; 95%IC: 0.25-1.12;* p *= 0.10).

**Conclusion:**

KAI can increase clinical effectiveness, improve quality of life, alleviate ADRs, and prolong survival time in advanced colorectal (CRC) patients receiving chemotherapy.

## 1. Introduction

Colorectal cancer (CRC), including colon and rectal cancers, is the third most common diagnosed malignancy and the second most common cause of cancer-related deaths in the world [1]. Over 1.8 million new CRC cases and 881,000 deaths were estimated to occur in 2018 [1]. The incidence and mortality rates of CRC have been increasing in China and have become a major public health problem in the country [2–6]. Moreover, the incidence rate of younger patients with CRC was rising [7]. Currently, the treatment for CRC mainly includes surgery, chemotherapy, radiotherapy, targeted therapy, immunotherapy, and comprehensive treatment [8]. For patients with an early stage, surgery is the main effective treatment. However, more than 50% patients were diagnosed until they enter into advanced stages; therefore, the operation was generally not suitable [9–12]. Although chemotherapy intervention is the mainstay of unresectable metastatic CRC treatment, many factors, such as lack of selectivity for tumor cells, insufficient drug concentration in tumor tissues, drug resistance, and systemic toxicity, affect the efficacy of chemotherapy and quality of patients life [5, 13]. Therefore, a more effective therapy is still necessary and urgent.

Traditional Chinese medicine (TCM), which is a promising alternative therapy for the treatment of CRC, has evolved over thousands of years in China and has been known to prevent tumorigenesis, minimize toxicity, reinforce the treatment effect, improve quality of life, and revert multidrug resistance[14–17]. Increasing evidence [18–21] has demonstrated that TCM combined with chemotherapy could significantly increase efficacy, improve quality of life, and alleviate the toxicity of chemotherapy. The Kangai injection (KAI), a TCM, consisting of* Ginseng*,* Astragali radix and Kushen*, has been widely applied as auxiliary treatment for multiple tumor in the clinic, and it has been demonstrated that it can enhance immunity, strengthen the effect of chemotherapy, improve quality of life, and alleviate adverse drug reactions (ADRs) [22, 23]. However, KAI as an adjuvant drug to increase efficacy, improve quality of life, and alleviate the ADRs of CRC patients receiving chemotherapy has not been systemically reviewed thus far.

Therefore, a systematic review and meta-analysis were conducted to compare the clinical effective rate, quality of life, ADRs, and survival time of patients who were treated with KAI combined with chemotherapy versus those who were treated with chemotherapy alone.

## 2. Methods

### 2.1. Databases and Search Strategy

Various databases were searched from the database inception to March 2019, including English databases PubMed, the Cochrane Library, Web of Science and the Chinese databases China National Knowledge Infrastructure (CNKI), the VIP information resource integration service platform (VIP), and Wanfang Data knowledge service platform (Wanfang Data). The English terms used were (“colorectal cancer” OR “colorectal carcinoma” OR “carcinoma of large intestine” OR “colorectal neoplasm”) AND (“kangai injection” OR “kang'ai injection” OR “traditional Chinese medicine” OR “Chinese medicine” OR “Chinese herbs”) AND (“chemotherapy” OR “chemotherapeutic” OR “infusion chemotherapy” OR “chemical therapy” OR “chemotherapy combined”). The Chinese terms used were “kangai zhusheye”, “kangai zhusheji”, “zhongyao”, “dachangai”, “jiechangai”, “jiezhichangai”, “zhichangai” and “lianhehualiao”. Two reviewers (Siqi Huang and Shaofan Zhang) independently retrieved articles from the databases using the same search terms. All identified literatures were screened after duplication checking with EndNote X8.1.

### 2.2. Inclusion Criteria

Included studies met the following criteria: (1) patients were diagnosed and confirmed as advanced CRC; (2) randomized controlled trial (RCT); (3) KAI was the only Chinese patent medicine used in RCTs; (4) KAI combined with chemotherapy treatment served as the experimental group, and chemotherapy treatment alone served as control group; (5) two or more of the following outcomes were measured: clinical effective rate, performance status (the Karnofsky performance scale, KPS), ADRs including nausea and vomiting, diarrhea, leukopenia, thrombocytopenia, liver dysfunction, renal dysfunction, peripheral neuropathy, and survival time.

### 2.3. Exclusion Criteria

Studies were excluded based on the following criteria: (1) reviews, meeting abstracts, and animal experiments; (2) the clinical stage of CRC was not advanced stage or was not clear; (3) patients had other tumors in addition to CRC; (4) treatment was combined with other traditional Chinese herbs; (5) incomplete or missing data; (6) the presence of less than two of the abovementioned outcomes.

### 2.4. Quality Assessment and Data Extraction

The quality of the included studies was evaluated according to the criteria of the Cochrane Handbook for Systematic Reviews of Interventions [24]. Selection bias (random sequence generation and allocation concealment), performance bias (blinding of participants and personnel), detection bias (blinding of outcome data), attrition bias (incomplete outcome data), reporting bias (selective reporting), and other bias were assessed according to the criteria of the Cochrane Handbook for Systematic Reviews of Interventions. The three biases of judgment were low risk, high risk, and unclear. Two reviewers (PengJi Yi and PanPan Xu) independently screened and extracted data from the texts. Any disagreements were ultimately resolved by two senior authors (Dan Mao and Weijun Peng). The following information was extracted: authors, year of publication, median age, sample size, gender, study design, KAI, details of intervention, and outcomes (tumor response, KPS, ADRs, and so on).

### 2.5. Statistical Analysis

(1) All the meta-analyses were performed using the Cochrane Collaboration software (RevMan 5.3). (2) The Odds Ratio (OR) or the Risk Ratio (RR) was used to analysis dichotomous variables such as clinical efficacy, KPS, and ADRs. If the 95% confidence interval (CI) did not include the value 1, the OR or RR point estimate was considered statistically significant at the* p *value less than 0.05. The weighted mean difference (WMD) was used to analysis continuous variables. If the 95% confidence interval (CI) did not include the value 0, the WMD point estimate was considered statistically significant at the* p* value less than 0.05. (3) The heterogeneity of the data assessed by the Chi^2^ test and* I*^2^ test. The combined data were considered as having heterogeneity if* p *< 0.1 or* I*^2^ > 50% and a random-effects model was used; otherwise, a fixed-effects model was used. Sensitivity was used for the heterogeneity. (4) Begg's test and funnel plot analyses were used to determine the publication bias of articles by Stata12.0. A two-tailed* p* value less than 0.05 was considered significant.

## 3. Results

### 3.1. Search Results and Study Characteristics

**Figure 1:**
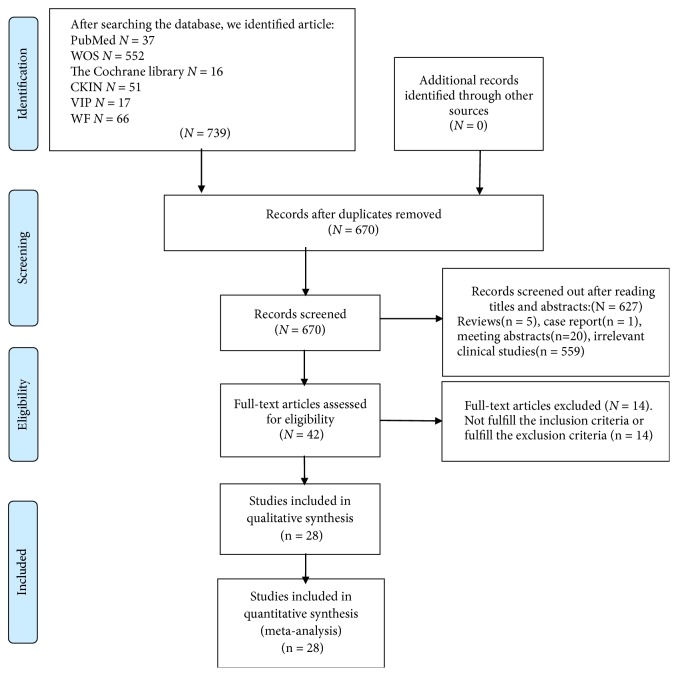
PRISMA flow diagram.

A total of 739 publications were identified using the predefined search strategy through several electronic databases. After excluding duplicates by EndNote X8, 670 studies were selected for analysis of the title and abstracts, and 43 studies were selected for through text reading. Fourteen studies did not meet the inclusion criteria or met the exclusion criteria: 5 articles included other tumors in addition to CRC; 3 articles included clinical stages of CRC that were not advanced stage; 1 article included other Chinese medicine; and 5 articles had no target outcomes. Finally, twenty-eight studies [25–52] were identified to meet the inclusion criteria. In this study, there were a total of 2310 cases, including 1207 cases for chemotherapy combined with KAI and 1103 cases for chemotherapy alone (Figure 1). The baseline characteristics for each study are shown in Table 1.

### 3.2. Risk of Bias

**Figure 2:**
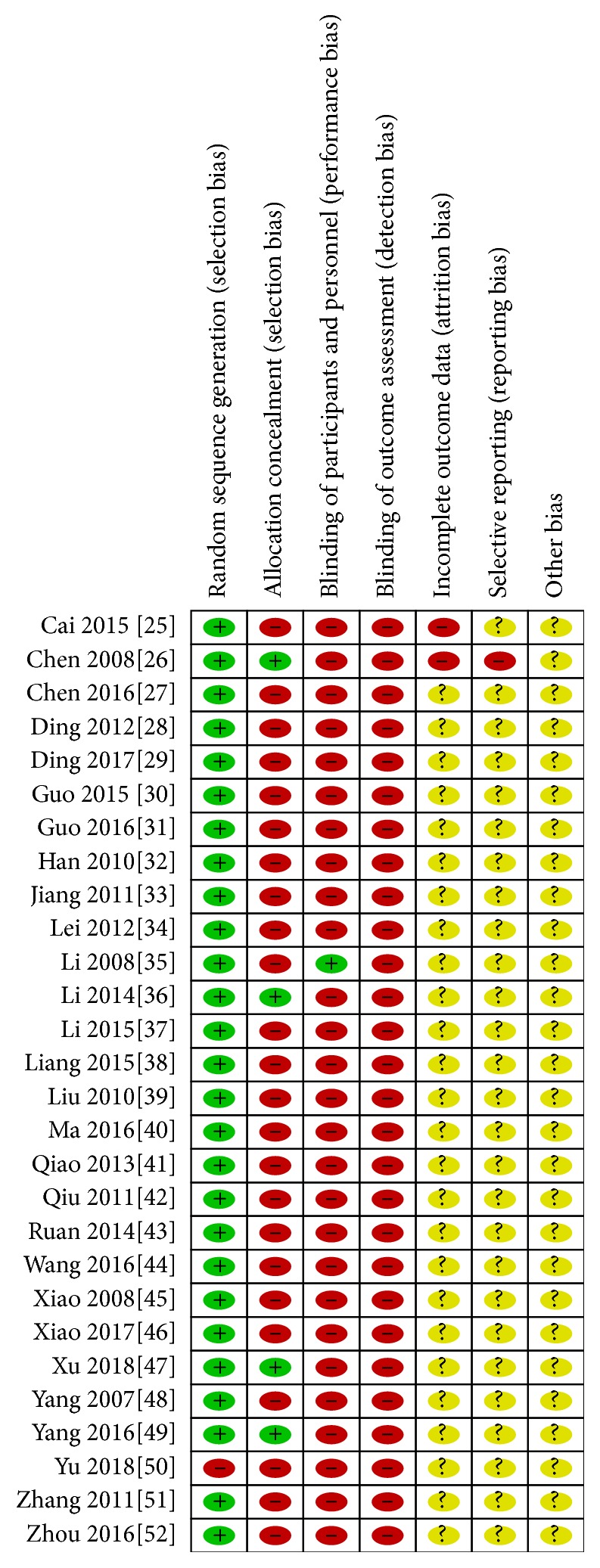
Risk of bias summary.

**Figure 3:**
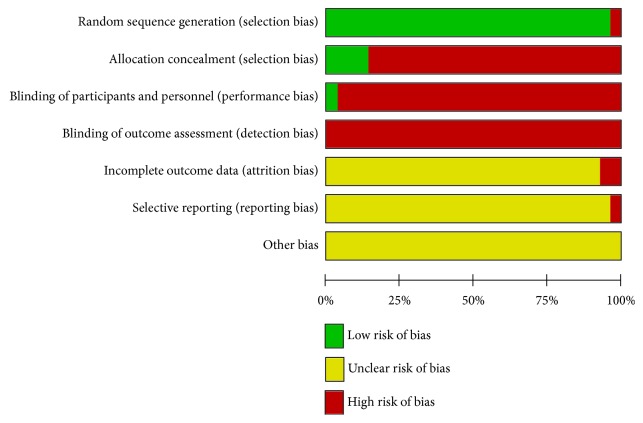
Risk of bias.

The quality of the included studies was generally low. Only eleven studies described the special randomization method, although all studies mentioned the random sequence generation. Four of the twenty-eight studies mentioned allocation concealment. One study provided information about the blinding of participants and personnel, blinding of outcome assessment, and selective reporting. The risk of bias is shown in Figures 2 and 3.

### 3.3. Results

#### 3.3.1. Tumor Response

**Figure 4:**
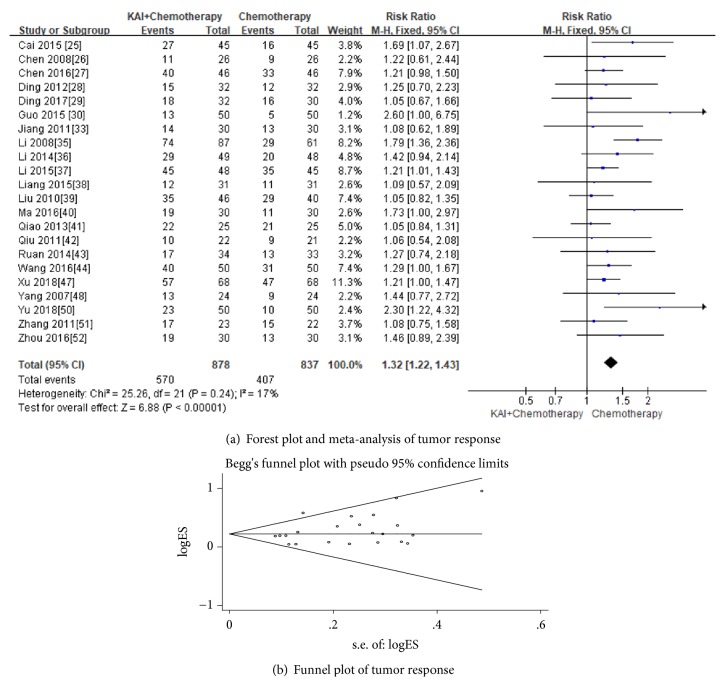


In this meta-analysis, 22 [25–31, 33, 35–44, 47, 48, 50–52] studies that assessed complete response (CR) or partial response (PR) in 1715 patients showed significant differences between KAI combined with chemotherapy group and chemotherapy alone group (Risk Ratio (RR) =1.32; 95% CI: 1.22-1.43;* p*<0.00001) (Figure 4(a)). There was no heterogeneity between two groups (Chi^2^=25.26, p =0.24; I^2^ =17%) and fixed-effects model was used to analyse the data. No significant publication bias was detected by Begg's test (*p*=0.099) (Figure 4(b)).

#### 3.3.2. Performance Status

**Figure 5:**
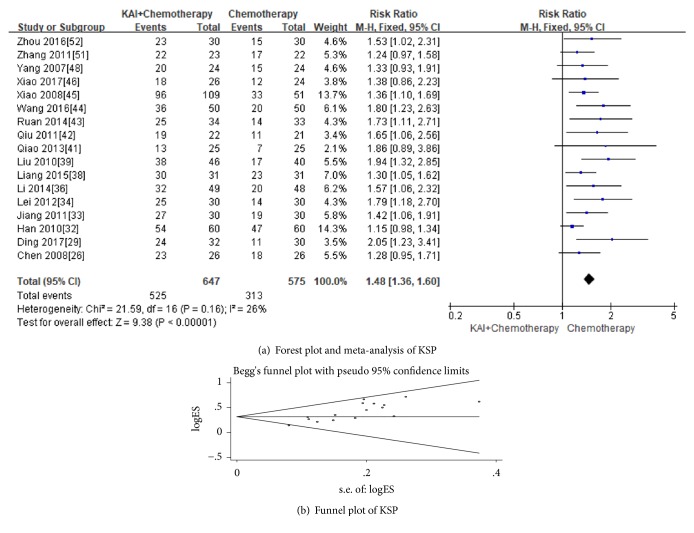


There were 17 [26, 29, 32–34, 36, 38, 39, 41–46, 48, 51, 52] studies that assessed KPS scores in the meta-analysis. No significantly heterogeneity between two groups (Chi^2^ = 21.59,* p* = 0.16; I^2^ = 26%) and we used fixed-effects models to analysis. The results showed that there was a statistically significant difference between the two groups (Risk Ratio (RR) =1.48; 95% CI: 1.36-1.60;* p*<0.00001). This result indicated that KAI combined with chemotherapy significantly improved KPS when compared with chemotherapy alone (Figure 5(a)). The Begg's test detected publication bias (*p *= 0.001) (Figure 5(b)).

#### 3.3.3. Adverse Drug Reactions

**Figure 6:**
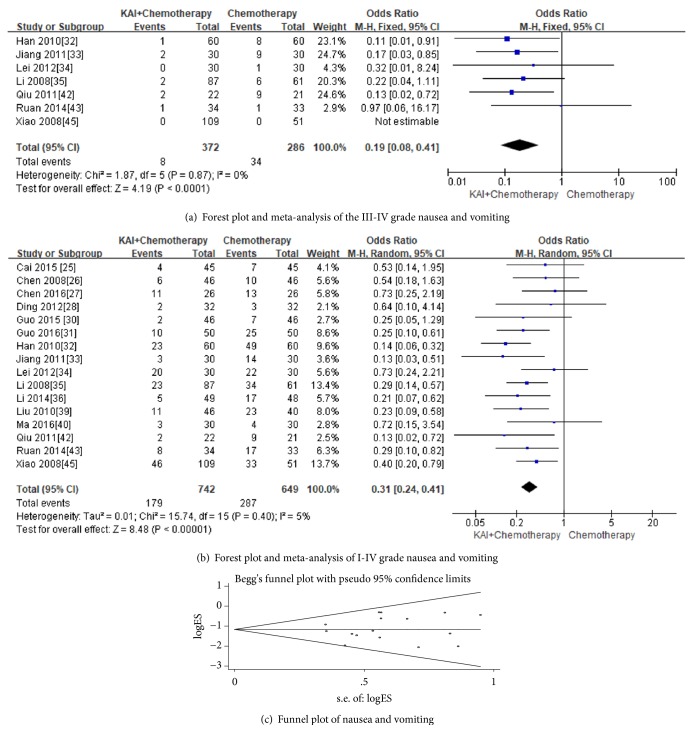


Two stages of nausea and vomiting were reported in these studies. As for toxicity grades III-IV of nausea and vomiting, seven trails [32–35, 42, 43, 45] including 658 cases were assessed. No heterogeneity was found (Chi^2^ =1.87, P = 0.87; I^2^ = 0%), so fixed-effect model was applied to analyse the data. The results showed that KAI can alleviate III-IV nausea and vomiting caused by chemotherapy, despite the effect which was moderate (Odds Ratio (OR) =0.19; 95%CI: 0.08-0.41;* p*<0.00001) (Figure 6(a)). Sixteen trials [25–28, 30–36, 39, 40, 42, 43, 45] with 1391 cases (741 cases of experimental group and 649 cases of control group) provided the results for nausea and vomiting. The fixed-effect model was applied to analyse the data (Chi^2^ =15.74, P = 0.40; I^2^ = 5%). The results indicated that there was a statistically significant difference between the two groups and KAI combined with chemotherapy can significantly alleviate nausea and vomiting when compared with chemotherapy alone (Odds Ratio (OR) =0.31; 95%CI: 0.24-0.41;* p*<0.00001) (Figure 6(b)). No publication bias was detected by Begg's test (*p *= 0.594) (Figure 6(c)).

**Figure 7:**
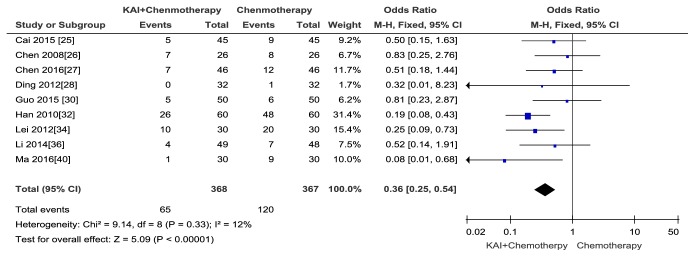
Forest plot and meta-analysis of diarrhea.

Nine studies [25–28, 30, 32, 34, 36, 40] that assessed 735 patients (368 patients in the experimental group and 367 patients in the control group) reported diarrhea. The fixed-effect model was applied to analysis because there was no heterogeneity (Chi^2^ =9.14,* p* = 0.33; I^2^ = 12%). The results indicated that there was a statistically significant difference between the two groups and KAI combined with chemotherapy notably improved diarrhea compared with chemotherapy alone (Odds Ratio (OR) =0.36; 95% CI: 0.25-0.52;* p*<0.00001) (Figure 7). No publication bias was detected by Begg's test (*p *= 0.864).

**Figure 8:**
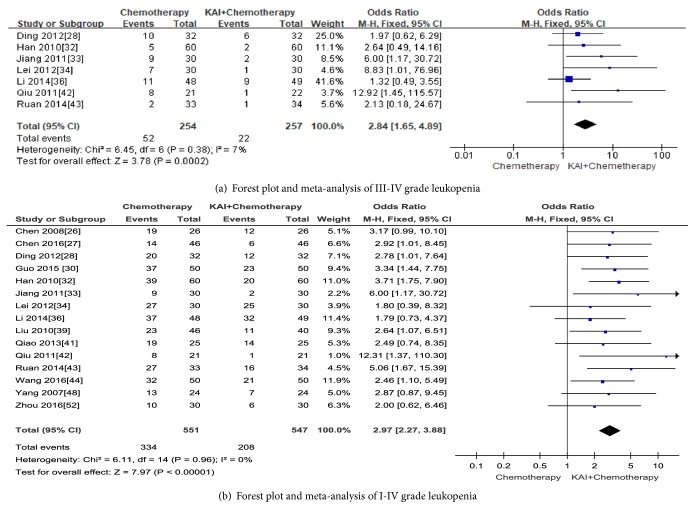


Senven studies [28, 32–34, 36, 42, 43] that evaluated 511 cases reported leukopenia with the toxicity grades III-IV. The result showed that KAI combined with chemotherapy improve leukopenia which caused by chemotherapy (Odds Ratio (OR) =2.84; 95% CI:1.65-4.89;* p*<0.0002). The fixed-effect model was performed to analysis because there was no significant heterogeneity ((Chi^2^ =6.45,* p*= 0.38; I^2^ = 7%) (in Figure 8(a)). Fifteen studies [26–28, 30, 32–34, 36, 39–44, 48, 52] that assessed 1098 patients reported leukopenia with the toxicity grades I-IV. The difference was statistically in favour of KAI combined with chemotherapy (Odds Ratio (OR) =2.97; 95% CI:2.27-3.88;* p*<0.00001), with no heterogeneity between two groups (Chi^2^ =6.11,* p*= 0.96; I^2^ = 0%), so the fixed-effect model was used for analysis (in Figure 8(b)). No significant publication bias was detected by Begg's test (*p*= 0.224).

**Figure 9:**
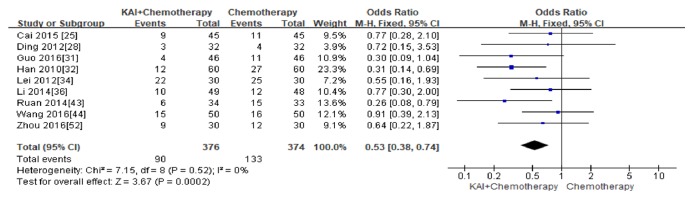
Forest plot and meta-analysis of thrombocytopenia.

Nine studies [25, 28, 31, 32, 34, 36, 43, 44, 52] that assessed 750 patients reported thrombocytopenia. The difference was statistically in favour of KAI combined with chemotherapy (Odds Ratio (OR) =0.53; 95% CI: 0.38~0.74;* p*<0.0002), with no significant heterogeneity between two groups (Chi^2^ =7.12,* p*= 0.42; I^2^ = 0%), so the fixed-effect model was used for analysis (Figure 9). No publication bias was detected by Begg's test (*p *= 0.893).

**Figure 10:**
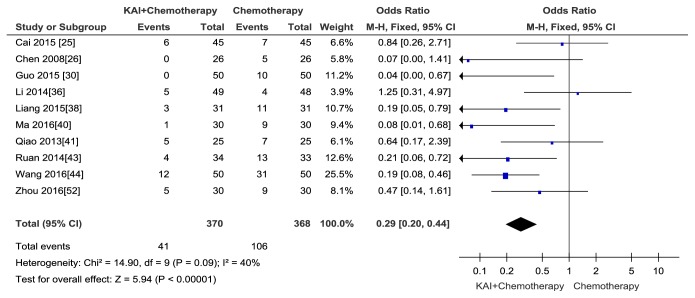
Forest plot and meta-analysis of liver dysfunction.

A total of ten publications [25, 26, 30, 36, 38, 40, 41, 43, 44, 52] with 738 patients reported liver dysfunction. No heterogeneity between two groups was observed (Chi^2^ =14.90, P = 0.09; I^2^ = 42%). The fixed-effects model was adopted to analyse data. The results suggested that there was a statistically significant difference between experimental and control groups and KAI combined with chemotherapy notably relived liver toxicity of chemotherapy when compared with chemotherapy alone (Odds Ratio (OR) =0.29; 95% CI: 0.20~0.44;* p*<0.00001(Figure 10)). No publication bias was detected by Begg's test (*p*= 0.326).

**Figure 11:**
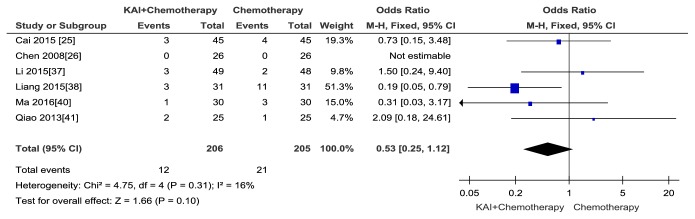
Forest plot and meta-analysis of renal dysfunction.

A total of six [25, 26, 37, 38, 40, 41] publications with 411 patients reported renal dysfunction. No heterogeneity between two groups was observed (Chi^2^ =4.75,* p* = 0.31; I^2^ = 16%) so the fixed-effects model was adopted to analyse data. The results suggested that there was no statistically significant difference between the experimental and control groups, and KAI combined with chemotherapy did not significantly relive the renal toxicity of chemotherapy when compared with chemotherapy alone (Odds Ratio (OR) =0.53; 95% CI: 0.25-1.12;* p *= 0.10) (Figure 11). No publication bias was detected by Begg's test (*p*=0.348).

**Figure 12:**
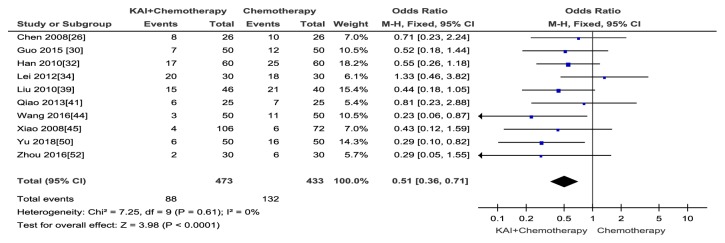
Forest plot and meta-analysis of neurotoxicity.

Neurotoxicity was reported in ten [26, 30, 32, 34, 39, 41, 44, 45, 50, 52] trails that contained 906 patients. KAI combined with chemotherapy was associated with a better protective effect against chemotherapy than chemotherapy alone, and the result was statistically significant (Odds Ratio (OR) = 0.51; 95% CI: 0.36-0.71;* p*= 0.0004). There was no heterogeneity, and the data were analysed by fixed-effects model (Chi^2^ = 7.25,* p*= 0.61; I^2^ = 0) (Figure 12). No publication bias was detected by Begg's test (*p *= 0.326).

#### 3.3.4. Immune Function

**Figure 13:**
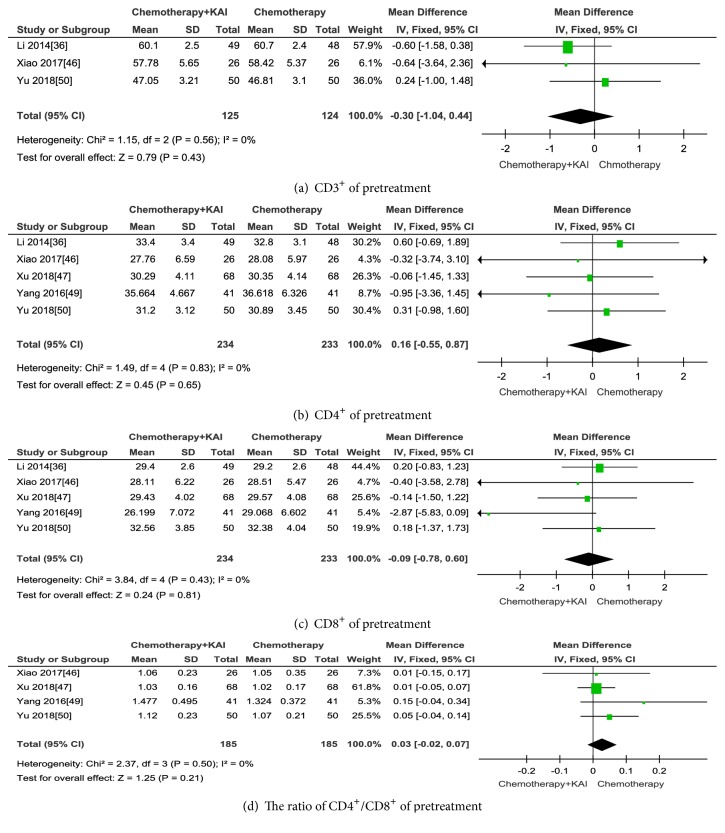


A total of five studies [36, 46, 47, 49, 50] reported immune function. Three trials [36, 46, 50] reported CD3^+^, five trials [36, 46, 47, 49, 50] reported CD4^+^, four trials [46, 47, 49, 50] reported CD8^+^, and three trials [36, 46, 50] reported the ratio of CD4^+^/CD8^+^. There was no significant difference in the pretreatment levels of CD3^+^, CD4^+^, CD8^+^, and the ratio of CD4^+^/CD8^+^ cells between the KAI combined with chemotherapy group and chemotherapy alone group (CD3^+^: MD=-0.30; 95% CI: -1.04-0.44;* p*=0.43.CD4^+^: MD=0.16; 95% CI: -0.55-0.87;* p*=0.65.CD8^+^: MD=-0.09; 95%IC: -0.78-0.60;* p=*0.8; the ratio of CD4^+^/CD8^+^: MD=0.03; 95% CI: -0.02-0.07;* p=*0.21) (Figures 13(a)–13(d)). A fix-effect model was used to analyse the data because heterogeneity was not observed (CD3^+^, CD4^+^, CD8^+^, and the ratio of CD4^+^/CD8^+^: I^2^ = 0).

**Figure 14:**
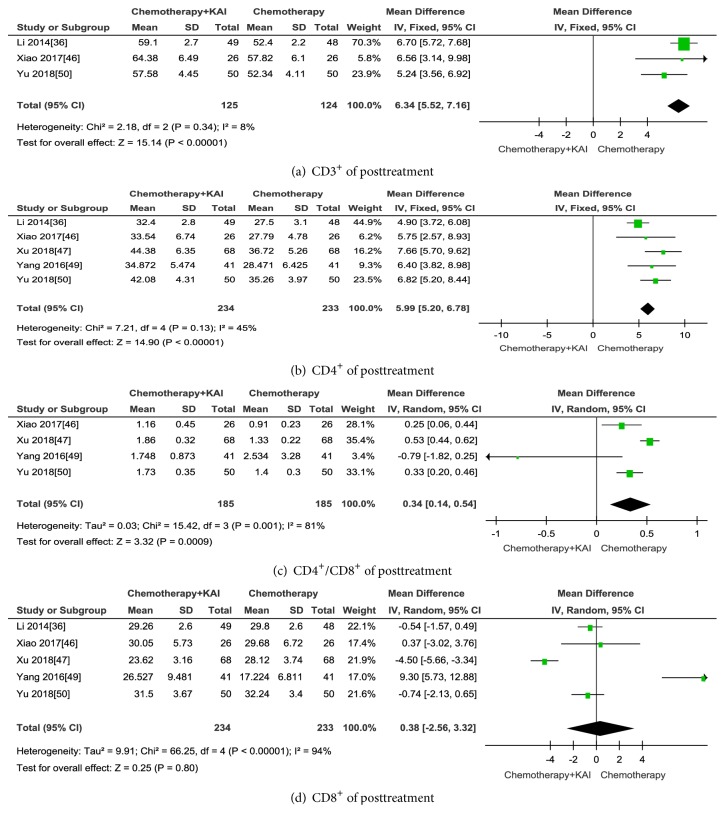


After treatment, the results indicated that KAI combined with chemotherapy can significantly increase the level of CD3^+^ (CD3^+^: MD=6.34; 95% CI: 5.52-7.16;* p *< 0.00001), CD4^+^ (CD4^+^: MD=-5.99; 95% IC: 5.20-6.78;* p *< 0.00001), and CD4^+^/CD8^+^ (CD4^+^/CD8^+^: MD=0.34; 95%CI: 0.14-0.54;* p *< 0.0009) when compared with chemotherapy alone (Figures 14(a)–14(c)). However, the CD8^+^ level was not significantly different between the two groups (CD8^+^: MD=0.38; 95% CI: -2.56-3.32;* p *= 0.80) (Figure 14(d)). Random-effect model was used to analyse data because heterogeneity of the ratio of CD4^+^/CD8^+^ (Chi^2^ =15.42,* p*= 0.001; I^2^ = 81%) and CD8^+^ (Chi^2^ =66.25,* p*< 0.00001; I^2^ = 94%) was high. For CD3^+^ and CD4^+^ fixed-effect model was used to evaluate data with moderate heterogeneity (CD3^+^: I^2^ = 8%; CD4^+^: I^2^ = 45%). No significant publication bias was observed (CD3^+^:* p*= 0.730; CD4^+^:* p*= 0.390; CD8^+^:* p*= 0.118; and the ratio of CD4^+^/CD8^+^:* p*= 0.130).

#### 3.3.5. Survival Rate

**Figure 15:**
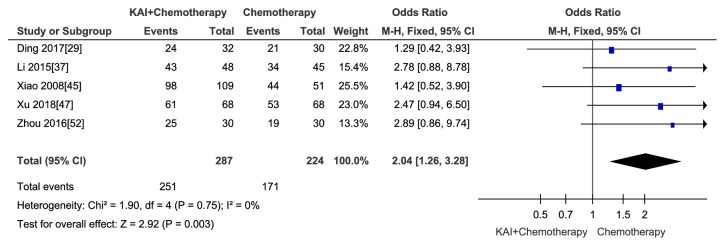
Forest plot and meta-analysis of survival rate.

In this meta-analysis, five studies [29, 37, 45, 47, 52] involving 511 participants reported the one-year survival rate. A fixed-effects model was used to analyse the data due to no heterogeneity (Chi^2^ =1.90,* p* = 0.75; I^2^ = 0%). The results indicated that there was a statistically significant difference between two groups (Odds Ratio (OR) =2.04; 95% CI: 1.26-3.28;* p *= 0.003) (Figure 15). No publication bias was detected by Begg's test (*p*= 0.294).

## 4. Discussion

CRC is one of the most common malignancies in the world [3]. More than 50% patients already entered end-stage when diagnosed with CRC and lost the chance of surgery [10]. Despite advances in treatment modalities, the adverse reactions like bone marrow suppression, gastrointestinal reactions, and multidrug resistance are still widespread and seriously affect cancer patient's quality of life even end treatment [10, 53].

TCM especially Chinese herbs medicines, as an important component of complementary and alternative medicine, has evolved over thousands of years in China with its own unique system of theories, diagnostic, and therapies [54]. KAI, a typical anticancer injection of TCM formula, mainly consists of* ginseng, astragali radix*, and* matrine.* (1) The major effective ingredients of* Ginseng* are* Ginsenoside (Ginsenoside Rg1, Ginsenoside Rb1, Ginsenoside Rg3, *and* Ginsenoside Rf*) and* Ginseng Polysaccharides*; these ingredients can improve immune functions and increase white blood cell (WBC) count after chemotherapy. (2) The major active ingredients of* astragali radix *is the root of the* Leguminous Plant Astragalus*, which has inhibitory effect on tumor cell proliferation and induces apoptosis. (3)* Matrine *and* oxymatrine*, two kinds of alkaloid form* Kushen*, have pharmacological activities to selectively kill tumor cells and inhibit tumor growth by changing the molecular sequence of the nucleic acids in cells [23, 55].

Accumulating clinical evidence demonstrated that KAI has played an important role in cancer therapy which can enhance the effect, improve quality of life, strengthen immune function, and reduce adverse reactions. A meta-analysis systematically evaluated the efficacy and safety of KAI combined with chemotherapy for treatment of breast cancer in 2018, and the result showed that KAI combined with chemotherapy for treating Chinese breast cancer can improve quality of life and minimize the adverse reactions [23]. A meta-analysis [56] and a randomized controlled trial [22] evaluated the efficacy and safety of KAI combined with chemotherapy for non-small-cell lung cancer (NSCLC), and the result demonstrated that KAI can enhance the therapy effect, improve quality of life, and reduce reverse reactions when combined with chemotherapy.

To the best of our knowledge, this study was the first time to systematically evaluate the synergistic and detoxifying effects of KAI therapy on advanced CRC patients receiving chemotherapy. This meta-analysis of twenty-eight RCTS including 2310 cases comparing the efficacy and safety of KAI combined with chemotherapy and chemotherapy alone. Our results suggested that KAI plays an important role in enhancing efficacy, improving quality of life, alleviating adverse drug reaction (ADRs), strengthening immune function, and prolonging survival time of CRC patients receiving chemotherapy.

The following limitations of this meta-analysis must be concerned. First, although we searched the PubMed, Web of Science, and the Cochrane Library databases, all of the included studies and all the subjects in the studies were Chinese. Further research is needed to assess the efficacy of KAI in other populations. Second, the quality of the studies included in our meta-analysis was poor. Although most trials performed randomization, four studies referred to allocation concealment, and only one was a double-blinded study. Moreover, all trials were carried out at single a centre. Third, publication bias was found in one of outcomes (KSP), so the results should be interpreted with caution. Fourth, the heterogeneity of the level of CD8^+^ and the ratio of CD4^+^/CD8^+^ were observed to be high. Sensitivity analysis did not eliminate heterogeneity. The high heterogeneity might be due to differences in sample size, patient age, tumor stage and grade, difference doses of KAI, and other factors among the studies. Finally, although the doses of KAI in most studies were the same (40 ml/day), there were still differences (50 ml/day, 60 ml/day). Large doses may favour better results which may result in publication bias.

## 5. Conclusion

KAI combined with chemotherapy can improve the quality of life, enhance clinical effectiveness rate of chemotherapy, and reduce the chemo-induced toxicity of chemotherapy treatment for advanced colorectal cancer patients. However, the outcomes were evaluated in a purely Chinese population, and the long-term, high-quality studies with a large sample size are needed to confirm the efficacy and tolerability of KAI in other populations.

## Figures and Tables

**Table 1:** * Notes. *E/C: experiment group and control group. F/M: female and male. N: not mentioned. ①: tumor response. ②: KPS. ③: nausea and vomiting. ④: diarrhea. ⑤: leukopenia. ⑥: thrombocytopenia. ⑦: liver dysfunction. ⑧: renal dysfunction. ⑨: neurotoxicity. ⑩: immune function. ⑪: survival time.

Study ID	Sample size (E/C)	Gender (F/M)	Median age(E/C)	Study design(E/C)	Intervention		KAI dosage (ml/day)	Outcomes
					E	C		
Cai 2015 [25]	45/45	49/41	50.24±2.4	Parallel-group	FOLFIRI+KAI	FOLFIRI	40	①③④⑥⑦⑧
Chen 2008 [26]	26/26	24/28	59	Parallel-group	FOLFOX4+KAI	FOLFOX4	60	①②③④⑤⑦⑧⑨
Chen 2016 [27]	46/46	34/58	61.2±11.7/62.1±11.4	Parallel-group	FOLFOX4+KAI	FOLFOX4	40	①③④⑤
Ding 2012 [28]	32/32	25/39	54/53	Parallel-group	FOLFOX4+KAI	FOLFOX4	40	①③④⑤⑥
Ding 2017 [29]	32/30	24/38	60/58	Parallel-group	XELOX+KAI	XELOX	40	①②⑪
Guo 2015 [30]	50/50	45/55	58.2±1.2/58.4±2.6	Parallel-group	FOLFOX4+KAI	FOLFOX4	60	①③④⑤⑦⑨
Guo 2016 [31]	46/46	41/51	59.7±1.5/59.9±1.2	Parallel-group	FOLFOX4+KAI	FOLFOX4	50	③⑥
Han 2010 [32]	60/60	45/75	52/51	Parallel-group	FOLFOX+KAI	FOLFOX	40	②③④⑤⑥⑨
Jiang 2011[33]	30/30	18/42	52.7/54.3	Parallel-group	FOLFIRI+KAI	FOLFIRI	40	①②③⑤
Lei 2011 [34]	30/30	25/35	N	Parallel-group	FOLFOX4+KAI	FOLFOX4	50	②③④⑤⑥⑨
Li 2008 [35]	87/61	65/83	55	Parallel-group	5FU+CF+KAI	5FU+CF	40	①②③
Li 2014 [36]	48/49	42/55	58.9±1.58/59.2±1.62	Parallel-group	FOLFIRI+KAI	FOLFIRI	40-60	①②③④⑤⑥⑦⑧⑩
Li 2015 [37]	45/48	41/52	57.13±7.05/56.72±7.24	Parallel-group	XELOX+KAI	XELOX	40	①⑪
Liang 2015[38]	31/31	24/38	53.8±6.4	Parallel-group	FOLFOX+KAI	FOLFOX	40	①②⑦⑧
Liu 2010[39]	46/40	32/54	65	Parallel-group	FOLFOX4+KAI	FOLFOX4	60	①②③⑤⑨
Ma 2016[40]	30/30	18/42	55±2.5	Parallel-group	mFOLFOX6+KAI	mFOLFOX6	40	②③④⑦⑧
Qiao 2015[41]	25/25	17/33	54.4/56.2	Parallel-group	FOLFOX+KAI	FOLFOX	40	①②⑤⑦⑧⑨
Qiu 2011[42]	21/22	16/27	52.7/56.9	Parallel-group	FOLFOX4+KAI	FOLFOX4	40	①②③⑤
Ruan 2014[43]	33/34	34/33	N	Parallel-group	XELOX+KAI	XELOX	40	①②③⑤⑥⑦
Wang 2016[44]	50/50	46/54	56.4±4.9/56.8±4.6	Parallel-group	FOLFOX+KAI	FOLFOX	50	①②⑤⑥⑦⑨
Xiao 2008 [45]	51/109	48/112	56/53.5	Parallel-group	FOLFOX+KAI	FOLFOX	40	②③⑨⑪
Xiao 2017 [46]	26/24	26/24	55	Parallel-group	mFOLFOX6+KAI	mFOLFOX6	50	②⑩
Xu 2018 [47]	68/68	63/73	56.5/57	Parallel-group	FOLFOX+KAI	FOLFOX	40	①⑩⑪
Yang 2007[48]	24/24	18/30	56	Parallel-group	FOLFOX+KAI	FOLFOX	50	①②⑤
Yang 2016[49]	41/41	44/38	71	Parallel-group	FOLFOX4+KAI	FOLFOX4	40	②⑩
Yu 2018[50]	50/50	33/67	55.4±5.8/54.6±6.1	Parallel-group	FOLFOX+KAI	FOLFOX	60	①⑨⑩
Zhang 2011[51]	22/23	15/30	42-71/45-73	Parallel-group	L-OHP+5FU+KAI	L-OHP+5FU	40	①②
Zhou 2011[52]	30/30	42/19	60.0±1.5/61.0±1.0	Parallel-group	FOLFOX+KAI	FOLFOX	60	①②⑤⑥⑦⑨⑪
